# Research on precise segmentation model of thyroid nodules based on deep learning

**DOI:** 10.1371/journal.pone.0358892

**Published:** 2026-10-08

**Authors:** Qingpeng Zhang, Qinqin Xiao, Jiacheng Wu, Guangming Shao

**Affiliations:** 1 School of Medical Information Engineering, Anhui University of Chinese Medicine, Hefei, Anhui, China; 2 College of Automation Engineering, Nanjing University of Aeronautics and Astronautics, Nanjing, Jiangsu, China; RLBCAU: Rani Lakshmi Bai Central Agricultural University, INDIA

## Abstract

Thyroid nodules are a prevalent condition in clinical practice, typically identified via ultrasound imaging with visual assessment by physicians. However, the ambiguous features of malignant nodules, their proximity to surrounding tissues, image noise, and unclear boundaries can lead to variability in diagnostic outcomes among different practitioners. In this study, we initially employed nine established models to segment ultrasound images from two publicly available datasets, TN3K and DDTI. Evaluation metrics including IoU, F1-score, accuracy (Acc), specificity (Spec), and precision (Pre) were utilized to assess the segmentation performance of these models. The experimental results indicate that, given the current data scale, the overall segmentation performance of each model is relatively low. Analysis suggests this is primarily due to the limited number of samples in the medical imaging dataset. During training, the model struggles to fully learn the discriminative features of the lesion area. Particularly during the encoder’s down-sampling stage, the feature expression ability is constrained, affecting the model’s ability to accurately recognize lesion locations. To address this, we enhanced the classic model by incorporating the Residual Network ResNet34, constructing a new thyroid nodule segmentation model. The improved model demonstrated enhanced segmentation performance, with most IoU values on the TN3K dataset exceeding 0.8. However, models trained on the DDTI dataset rarely achieved high performance. Further analysis attributes this performance disparity to differences in the number of training samples. To further enhance model performance, we initialized the improved model with pre-trained ResNet34 weights from the ImageNet dataset, resulting in significant performance improvements. Notably, Res34-PAN performed best across both datasets. On the DDTI dataset, the model achieved an IoU of 0.8778, an F1-score of 0.9349, an accuracy of 0.9872, a specificity of 0.9928, and a precision of 0.9342. To further validate the model design’s rationale, we conducted a systematic ablation experiment. We selected commonly used network structures for encoders and used them as the encoders for nine segmentation models, comparing performance with and without the ImageNet pre-trained weights. The results further confirmed the effectiveness of the proposed method in the thyroid nodule segmentation task.

## Introduction

Thyroid nodules, resulting from the abnormal proliferation of thyroid cells [1], are a common condition, particularly among women and the elderly [2]. Research indicates that approximately 4% to 7% of adults have palpable thyroid nodules [3], but only about 8% to 16% of these cases progress to thyroid cancer [4]. The primary method for preoperative diagnosis is fine-needle aspiration cytology (FNAC) [5], which boasts an accuracy rate of 97.5% [6]. Thyroid-stimulating hormone (TSH) tests and ultrasound (US) are crucial in diagnosing and treating thyroid nodules, providing insights into thyroid function and the characteristics of malignant tumors. Technological advancements and the convenience of US have made it a popular diagnostic tool, capable of detecting ten times more nodules than traditional methods. However, despite its widespread use and ability to identify more nodules, US is less accurate than FNAC, leading to an increase in misdiagnoses in recent years [7].

In recent years, numerous scholars have dedicated themselves to researching accurate methods for diagnosing and treating thyroid nodules. A study by the Italian Minimally Invasive Thyroid Treatment Group suggested that thermal ablation could serve as a viable alternative to surgical intervention for patients with thyroid nodules [8]. In a comparative study conducted by Hisham Mehanna et al., it was observed that ultrasound elastography, when compared to traditional ultrasound-guided fine-needle aspiration cytology (US FNAC), marginally decreased the necessity for diagnostic thyroidectomies without significant additional benefits [9]. Luca Giovanella et al. proposed a modified diagnostic algorithm in thyroidology, leading to a reduction in FNAC procedures and diagnostic surgeries for patients with ambiguous thyroid nodules [10].

In computer science, the advancement of machine learning and deep learning has facilitated the convergence of medicine and engineering, paving the way for the advancement of precision medicine and intelligent healthcare. FCN [11], a variant of CNN [12], excels at recovering the category of each pixel in an image from abstract features, allowing for precise lesion localization. U-Net [13], with its “U”-shaped symmetric structure, effectively captures contextual information from medical images using an Encoder-Decoder framework. U-Net++ [14], a notable variant of U-Net, enhances segmentation performance by integrating multiple small U-Nets to fuse results from various depths. Since its introduction in 2015, ResNet [15] has significantly influenced the design of subsequent deep learning networks. Its distinctive residual learning and identity mapping techniques effectively mitigate gradient vanishing and explosion issues while deepening the model structure, thereby improving lesion localization.

Numerous image segmentation models are commonly utilized for segmenting various disease images, including cell nucleus, lung, and breast cancer segmentation. Wan Siti Halimatul Munirah Wan Ahmad and colleagues (2015) proposed a Gaussian-response based automatic threshold generation algorithm that demonstrates strong cross-device generalization for lung segmentation. Their approach achieved consistently high performance on both the JSRT and SH datasets, with most evaluation metrics exceeding 0.90 on JSRT [16]. Goceri (2024) employed a hybrid attention-aware network and adversarial network for cell nucleus segmentation. The hybrid attention block enhanced global information extraction and interrelations between local features, resulting in a minimum 3.7% increase in segmentation accuracy compared to contemporaneous methods [17]. Na et al. (2024) introduced an automatic prompt generator based on SAC [18] to guide cell nucleus segmentation effectively. They integrated low-rank attention into the SAM model, enhancing SAC’s adaptability across diverse segmentation tasks. Rezvani et al. (2025) proposed a hybrid approach using the FusionLungNet network, amalgamating the ResNet50 encoder, CAA, MFF, SR, and multiple decoders. Lung segmentation on the self-compiled LungSegDB dataset achieved an IoU of 98.04 [19]. Hasan et al. (2024) employed DeepLabV3+ for lung segmentation in chest X-ray images [20], yielding 97.42% accuracy, 93.49% Jaccard index, and 96.63% Dice coefficient, outperforming other models. Sulaiman et al. (2024) integrated the attention mechanism with U-Net, proposing the Attention U-Net model for precise depiction of breast tumors and boundary separation. On the BUSI dataset, the model achieved 0.98 accuracy, 0.97 precision, 0.90 recall, and 0.92 Dice coefficient [21]. Samudrala et al. (2024) utilized a hybrid model of DenseNet-121 and Att-PSPnet with an Attention Gate to mitigate noise interference, enhancing multiple evaluation metrics and achieving 94.68% accuracy [22]. Despite extensive research on medical image segmentation, studies on thyroid nodule segmentation remain limited. This study evaluated nine classic models, including U-Net, U-Net++, DeepLabV3 [23], DeepLabV3+ [24], FPN [25], LinkNet [26], MANet [27], PSPNet [28], and PAN [29], for segmenting thyroid nodule ultrasound images. Nine classical models were found to perform poorly in recognizing thyroid nodule lesions within the small-sample dataset. PAN exhibited superior performance among the classic models. Subsequently, classic models were enhanced by incorporating the ResNet34 residual network. Performance comparison revealed enhancements in all nine models, with notable improvements in LinkNet and MANet, and the optimized model Res34-PAN demonstrated the best performance.

## Materials and methods

### TN3K dataset

The dataset [30] comprising 3,493 2D ultrasound images of 2,421 patients from January 2016 to August 2020 was generously provided by Gong Haifan [31,32] and colleagues at Sun Yat-sen University. The dataset is divided into two segments: the training/validation set and the test set. The training/validation set comprises 2,879 ultrasound images, while the test set includes 614 ultrasound images. Sourced from real clinical cases, this dataset is highly regarded in the field of thyroid nodule segmentation. The official source provides a fixed division. For the experiments, the training/validation set is randomly split into a training set and a validation set at a 9:1 ratio, using a random seed of 42.

### DDTI dataset

The dataset [33] comprising 637 thyroid nodule ultrasound images in PNG format with pixel labels was obtained from the MICCAI 2020 TN-SCUI Thyroid Nodule Ultrasound Image Segmentation Competition [34], as curated by Pedraza et al. Notably, all images originate from a singular device. In this experiment, to evaluate the model’s performance and generalization ability, the dataset was randomly split into a training set, validation set, and test set in a ratio of 8:1:1, using a random seed of 42.

To address the inconsistent image sizes in the two datasets, OpenCV is employed to resize both the images and their corresponding annotations to 256 × 256. Once the images are read, if their pixel values fall within the range of [0, 255], normalization is applied, followed by standardization using ImageNet statistics. For the annotation masks, after reading, a single channel is selected for binary processing, where pixels with values ≥ 1 are set to 1, and all others are set to 0.

### ResNet34 residual network

The original U-Net encoder comprises multiple layers of convolution and pooling operations, resembling the simple structure of VGG-like convolutional networks. Despite its ease of training, the U-Net model struggles with limited feature representation and decreased gradient propagation efficiency as network depth increases. This limitation is particularly evident with small samples, where it fails to effectively model complex lesion features. In neural networks, both model depth and width significantly impact performance, and enhancing either can potentially boost outcomes. The introduction of the Residual Network (ResNet) has profoundly addressed issues like the vanishing gradient problem associated with increased model depth. As shown in Fig 1, ResNet employs multiple small convolution kernels instead of a single large one and connects outputs from upper and lower residual blocks to facilitate information reuse. This approach mitigates gradient dispersion and explosion issues caused by excessive convolution depth in traditional networks. The connection of residual blocks simplifies training and helps retain feature details in deeper convolutional layers. ResNet models are commonly categorized by their number of convolutional layers-152, 101, 50, 34, and 18-with those having 50 layers or more classified as deep residual networks.

**Fig 1:**
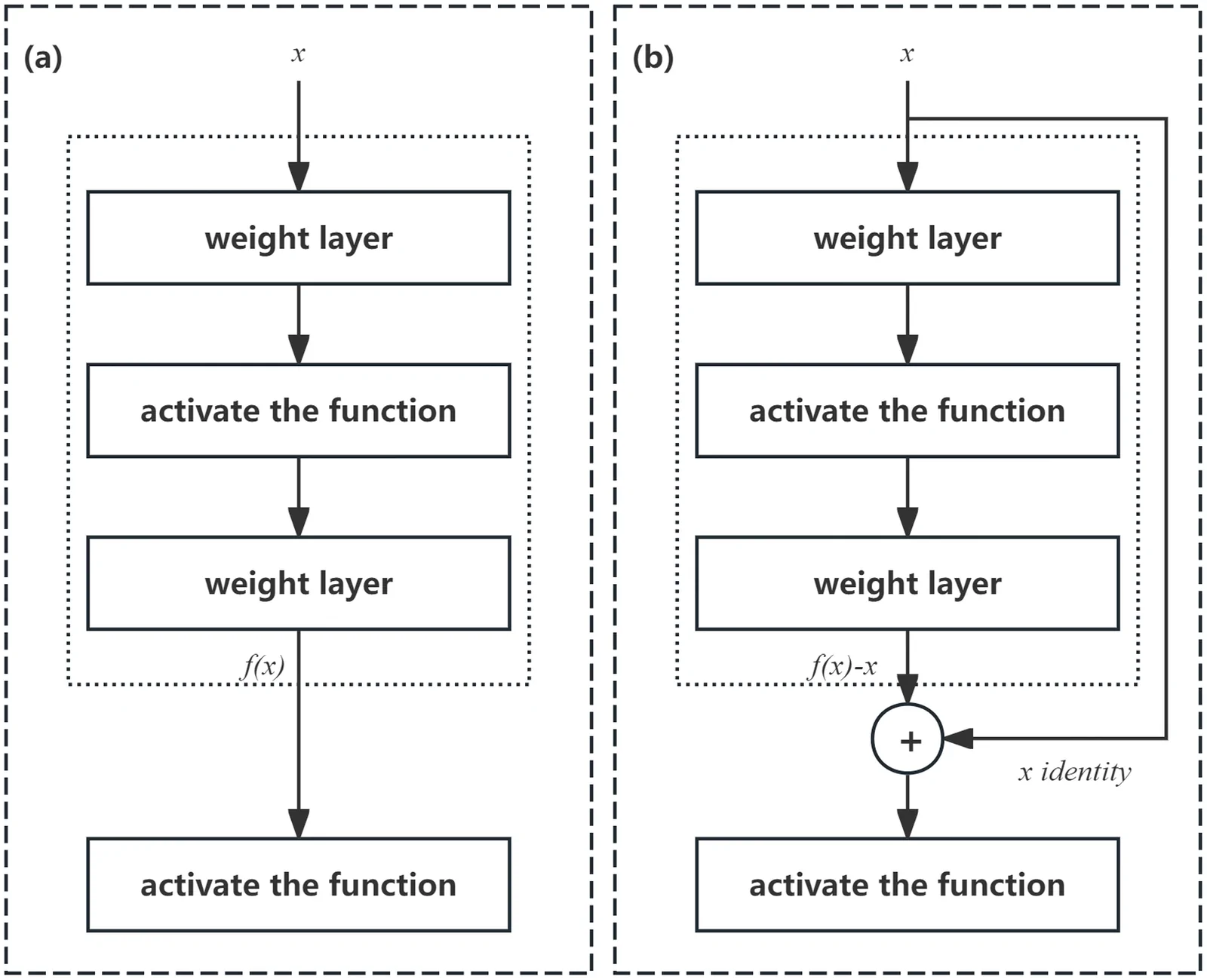
Standard block and residual block. (a) A standard block composed of two stacked weight layers with activation functions. (b) A residual block that adds an identity (shortcut) connection to the block output, enabling residual learning and improving gradient propagation in deeper networks.

In constructing the model, this paper employs the network parameters of ResNet34 as the encoder to enhance the classic model, taking into account both the number of model parameters and computational resources. As shown in Fig 2, the ResNet34 architecture, with its layers of residual blocks, is employed to improve the feature extraction process. Additionally, it incorporates the weights of ResNet34, which were trained on the large-scale ImageNet database, to optimize the improved model. By leveraging low- and mid-level visual features learned from this extensive natural image dataset and fine-tuning on the thyroid nodule ultrasound image dataset, the model’s feature transfer capability and segmentation accuracy are effectively enhanced, even with limited sample sizes. This approach helps mitigate the performance bottleneck caused by insufficient data samples.

**Fig 2:**
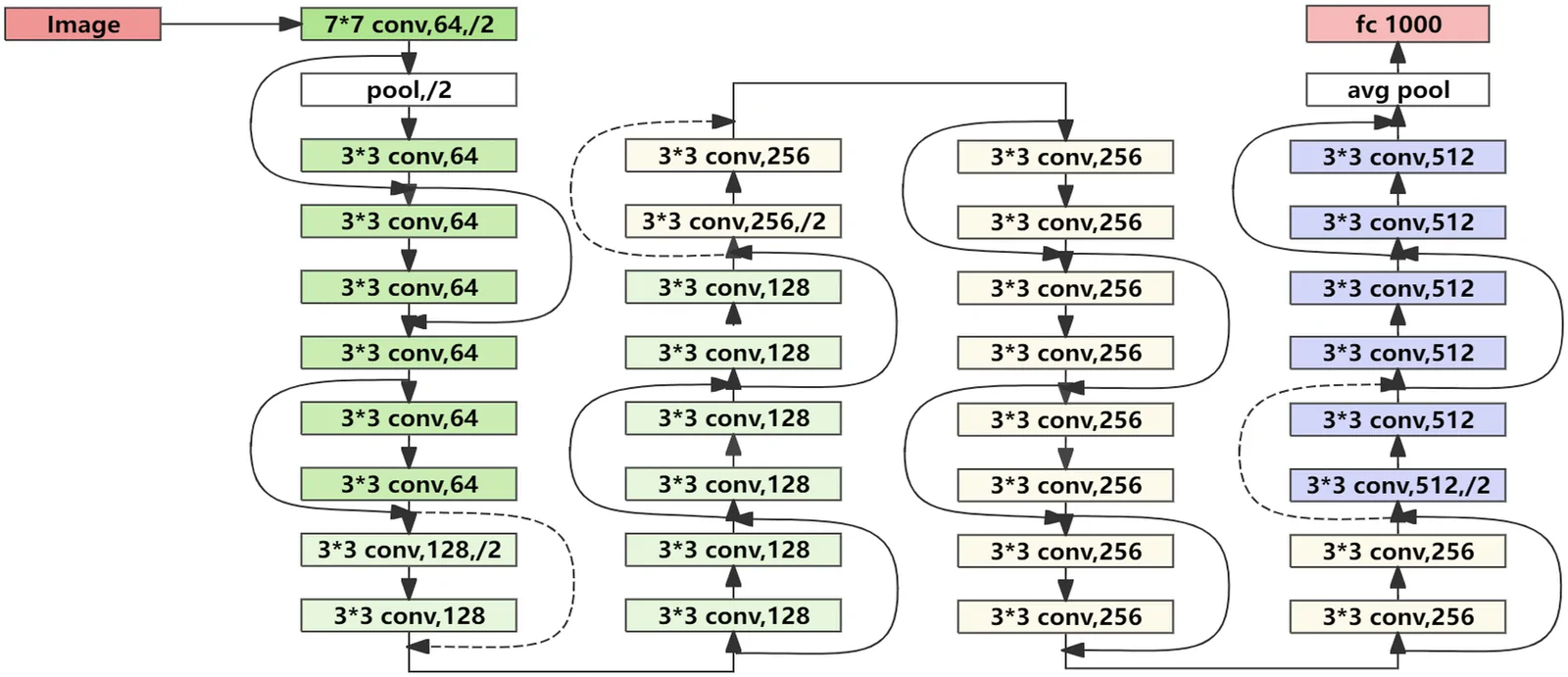
Schematic diagram of the ResNet34 architecture. ResNet34 is composed of an initial convolution and pooling layer followed by four stages of residual blocks (conv2_x–conv5_x). Each stage contains stacked residual blocks with identity (shortcut) connections, enabling deeper feature extraction and improved gradient propagation. In this study, ResNet34 (initialized with ImageNet-pretrained weights) is used as the encoder backbone for the improved segmentation models.

### PAN (Path Aggregation Network)

The PAN model is an enhancement of the FPN model, which propagates high-level semantic features using a top-down pyramid structure. As shown in Fig 3, PAN addresses the limitation of FPN by introducing a bottom-up pyramid structure following the FPN, facilitating the transfer of low-level localization features upwards. While FPN improves semantic information transfer, it lacks the ability to transfer localization information, necessitating further enhancement for improved model performance. This dual-path approach minimizes information loss, retains detailed information in feature maps, and enhances the model’s segmentation accuracy.

**Fig 3:**
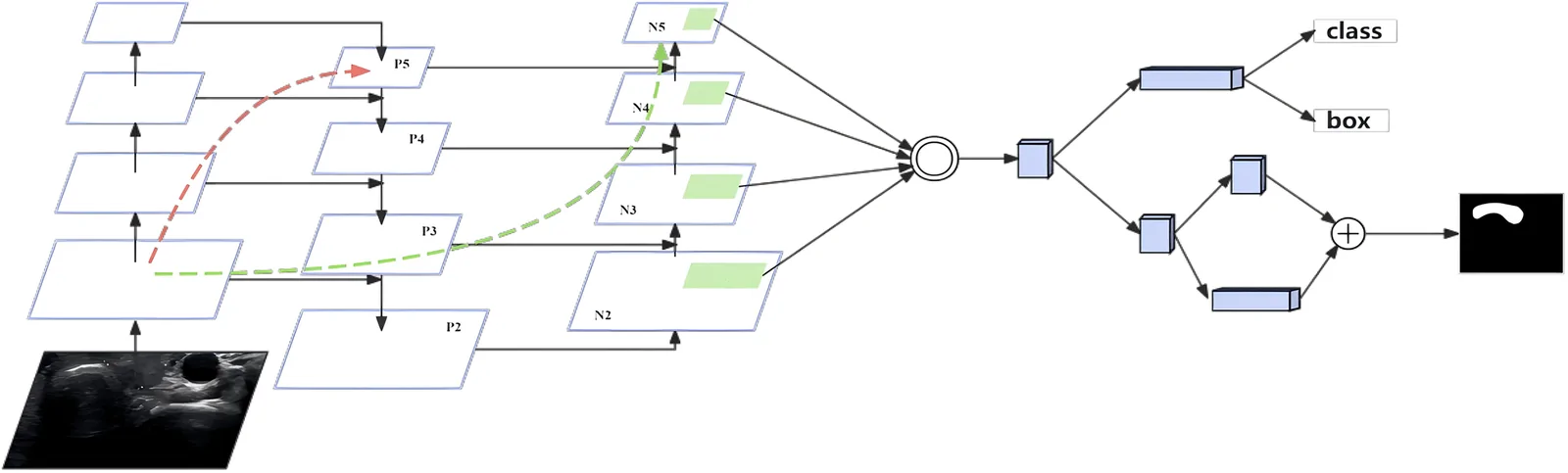
Architecture diagram of the PAN model. PAN (Path Aggregation Network) enhances the Feature Pyramid Network (FPN) by adding a bottom-up path augmentation after the top-down pyramid. The top-down pathway propagates high-level semantic features to lower-resolution feature maps, while the bottom-up pathway further aggregates low-level localization information upward. This dual-path feature fusion reduces information loss and improves the transfer of both semantic and localization cues, thereby enhancing segmentation performance.

### Model optimization

The research institute selected nine classic models in the semantic segmentation field, commonly applied in object detection and instance segmentation, all featuring Encoder-Decoder architectures. This study enhances these classic models by substituting the original Encoder component with ResNet34, while retaining the original Decoder structure. The construction of Res34-UNet and Res34-PAN serves as illustrative examples.

#### Res34-UNet.

The Res34-UNet model in this study follows a structure akin to the traditional U-Net, comprising an Encoder module and a Decoder module. Diverging from the U-Net architecture, ResNet34 is utilized for downsampling to capture features, supplanting the original downsampling mechanism. During upsampling, the Encoder of the conventional U-Net serves as the Decoder module, with Skip Connection facilitating the fusion of shallow and deep features between the Encoder and Decoder.

As shown in Fig 4, the Encoder module adopts the shallow residual network structure ResNet34 with 5 downsampling operations. Initially, the input image undergoes convolution processing, succeeded by a 3×3 max-pooling operation. Subsequently, it traverses four residual modules comprising varying numbers of residual blocks, processing the input feature vectors. These diverse residual modules effectively mitigate issues of gradient vanishing and network degradation encountered during downsampling in the conventional U-Net model. Lastly, a 2×2 max-pooling operation is applied to minimize noise interference in medical images.

**Fig 4:**
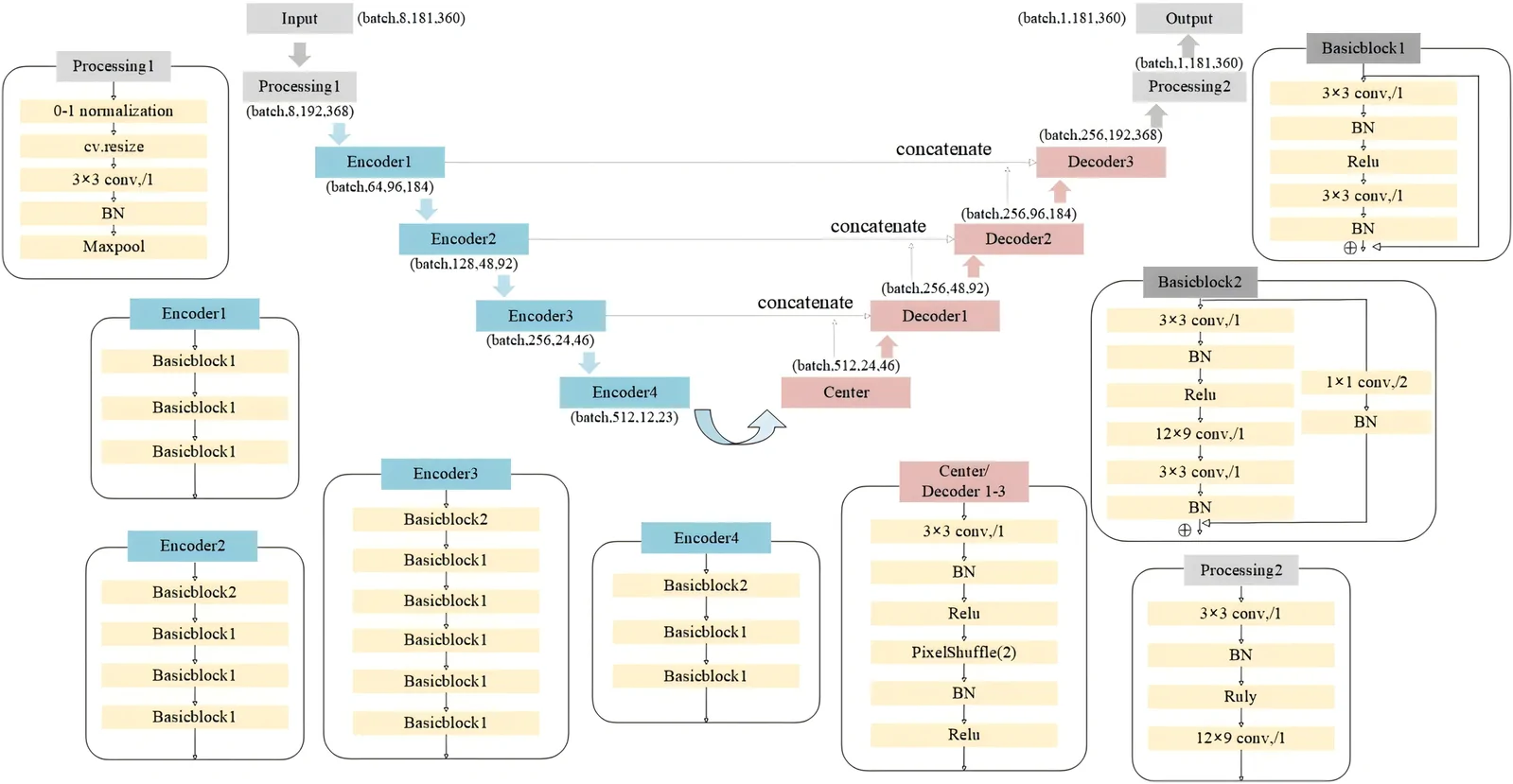
Res34-UNet network framework. The proposed Res34-UNet follows an encoder–decoder structure. The encoder replaces the original U-Net downsampling path with a ResNet34 backbone (including an initial convolution and max-pooling, followed by four residual stages) to extract multi-level features. The decoder performs symmetric upsampling to recover the spatial resolution, and skip connections fuse shallow detailed features with deep semantic features to improve boundary localization and segmentation accuracy.

The decoder of the traditional U-Net model employs the decoding module for upsampling, mirroring the number of upsampling iterations to match the downsampling steps in the encoder, both set at 5. Initially, the feature image undergoes two sequential processes involving the ReLu activation function and convolution. Subsequently, the U-Net model executes the upsampling operation to generate an output image identical in size to the original image.

#### Res34-PAN.

The Res34-PAN model introduced in this study retains the Encoder-Decoder structure of PAN, preserving both its Decoder and path aggregation mechanism, while exclusively modifying and reconstructing the Encoder of the original PAN. Like Res34-UNet, the primary design principle of Res34-PAN is to incorporate a residual network to improve feature extraction during the encoding phase, leaving the original decoding process intact.

As shown in Fig 5, during the encoding stage, Res34-PAN utilizes ResNet34 as the feature extraction network to conduct layer-by-layer downsampling and feature encoding on input thyroid nodule ultrasound images. ResNet34 is composed of several residual modules, each facilitating cross-layer feature transfer via residual connections. This design allows the network to maintain a stable information flow as its depth increases. The encoding method effectively extracts representative image features at various depths, offering multi-scale feature representations for the subsequent decoding stage.

**Fig 5:**
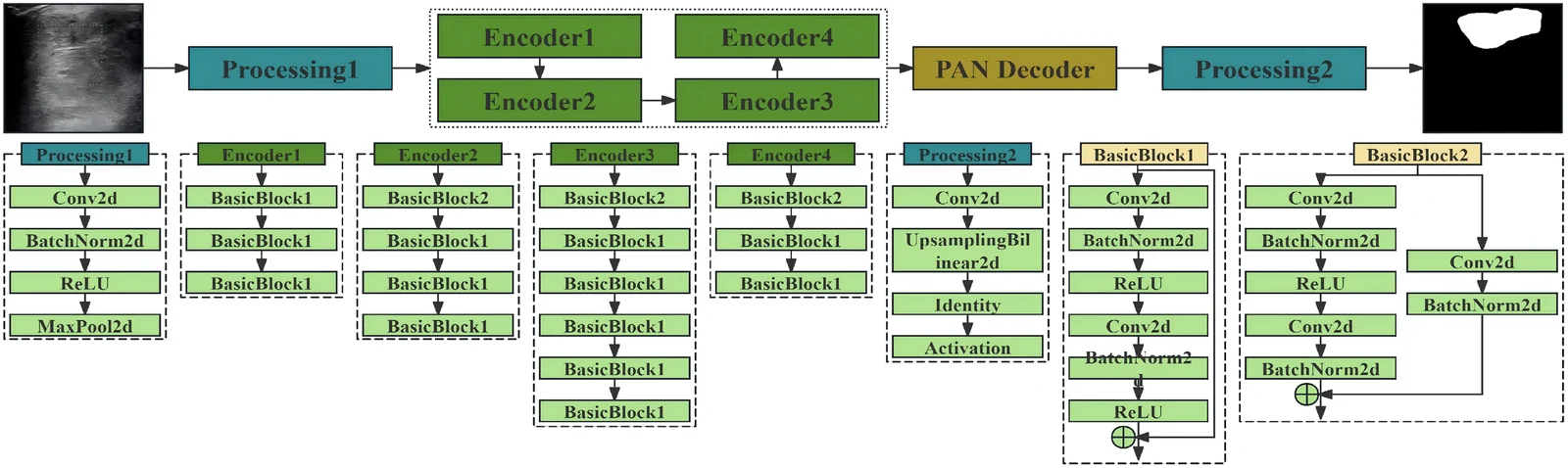
Res34-PAN network framework. Res34-PAN inherits the original PAN encoder–decoder topology and keeps the PAN decoder and path aggregation mechanism unchanged, while reconstructing the encoder by replacing it with ResNet34. The ResNet34 encoder performs progressive downsampling and multi-level feature encoding through residual blocks and shortcut connections, providing richer multi-scale feature representations. The PAN decoder then aggregates features via bidirectional information flow (top-down and bottom-up), fusing high-level semantic features with low-level spatial localization cues to generate the final pixel-wise thyroid nodule segmentation.

In the decoding stage, Res34-PAN retains the original PAN structure. By facilitating bidirectional information transfer both top-down and bottom-up, it effectively merges high-level semantic features with low-level spatial location information. Given that the features output during the encoding stage are richer in structural depth and semantic hierarchy, the decoder can achieve more comprehensive fusion and reconstruction of multi-scale features while adhering to the original design concept of PAN.

The primary structural enhancement of Res34-PAN is evident in the encoder layer. By substituting the original encoder with ResNet34, a residual learning mechanism is incorporated, while the decoder and overall network topology remain unchanged. This modification improves the model’s capability to capture multi-level features during the encoding phase, offering a more robust structural foundation for subsequent feature fusion and pixel-level prediction.

### Evaluation indicators and loss functions

To quantitatively assess the model’s performance in the thyroid nodule segmentation task, this study employs five common evaluation metrics: Intersection over Union (IoU), F1-score, Precision, Specificity, and Accuracy. IoU and F1-score gauge the overlap between the predicted segmentation region and the true label, serving as the primary metrics for evaluating segmentation effectiveness. Precision indicates the proportion of pixels correctly predicted as nodule regions, while Specificity assesses the model’s ability to identify non-nodule, or background, regions. Accuracy measures the proportion of correctly classified pixels relative to the total number of pixels, reflecting overall classification performance. All these metrics range from 0 to 1, with higher values signifying better segmentation performance.

Thyroid nodule segmentation is fundamentally a binary classification task at the pixel level. In this experiment, Dice Loss serves as the optimization objective. The network outputs pixel-level logits, which are transformed into predicted probabilities $p_{i}$ through the sigmoid activation function. The corresponding ground-truth binary label is denoted by $y_{i}$. For an image containing *N* pixels, the Dice coefficient is defined as follows:


$$\begin{array}{c} Dice=\frac{\text{2}\sum_{i=\text{1}}^{N}p_{i}y_{i}+\varepsilon }{\sum_{i=\text{1}}^{N}p_{i}+\sum_{i=\text{1}}^{N}y_{i}+\varepsilon } \end{array}$$ (1)

Accordingly, the Dice Loss is defined as:


$$\begin{array}{c} L_{Dice}=\text{1}-Dice \end{array}$$ (2)

where $p_{i}$ denotes the predicted probability of the i-th pixel, $y_{i}$ denotes the corresponding ground-truth binary label, *N* is the total number of pixels, and ε is a smoothing term used to avoid numerical instability when the denominator approaches zero. In this study, ε is set to 10^−6^.

## Results and discussion

The experimental environment of this experiment is shown in Table 1.

**Table 1:** Experimental environment configuration. This table lists the hardware and software environment used for model training and evaluation (CPU, GPU, Python, PyTorch, CUDA, and development tools).

Experimental Environment	Version
CPU	12th Gen Intel(R) Core(TM) i7-12700F 2.10 GHz
GPU	NVIDIA GeForce RTX 4090
Python	3.10.14
Pycharm	2022.2
CUDA	12.6
Pytorch	2.2.1 + cu121

The model training employs the Adam optimizer with an initial learning rate of 0.001. Throughout the training process, the batch size is maintained at 64, and the penalty coefficient is fixed at 0.000001, and iteration number interval of (60, 150) were specified. The optimal iteration number was determined based on the calculated loss function during training. By evaluating the model’s loss functions on the TN3K and DDTI datasets, an iteration number of 120 was chosen for the experiment. To mitigate sensitivity to the choice of the initial learning rate, we incorporated an adaptive learning-rate adjustment strategy during training. Specifically, we employed a ReduceLROnPlateau scheduler to monitor the validation loss; if no improvement was observed for 10 consecutive epochs, the learning rate was reduced by a factor of 0.5.

Introducing an adaptive learning rate adjustment mechanism mitigates the impact of the initial learning rate configuration on experimental outcomes. Initially, a relatively high learning rate expedites the model’s convergence towards the optimal solution during training. As training progresses, the algorithm dynamically adjusts the step size of parameter updates based on gradient information, progressively decreasing the learning rate to avert convergence to local optima. As shown in Fig 6, the optimized model incorporating ResNet34 weights from ImageNet converges more quickly and exhibits greater stability in its fluctuations.

**Fig 6:**
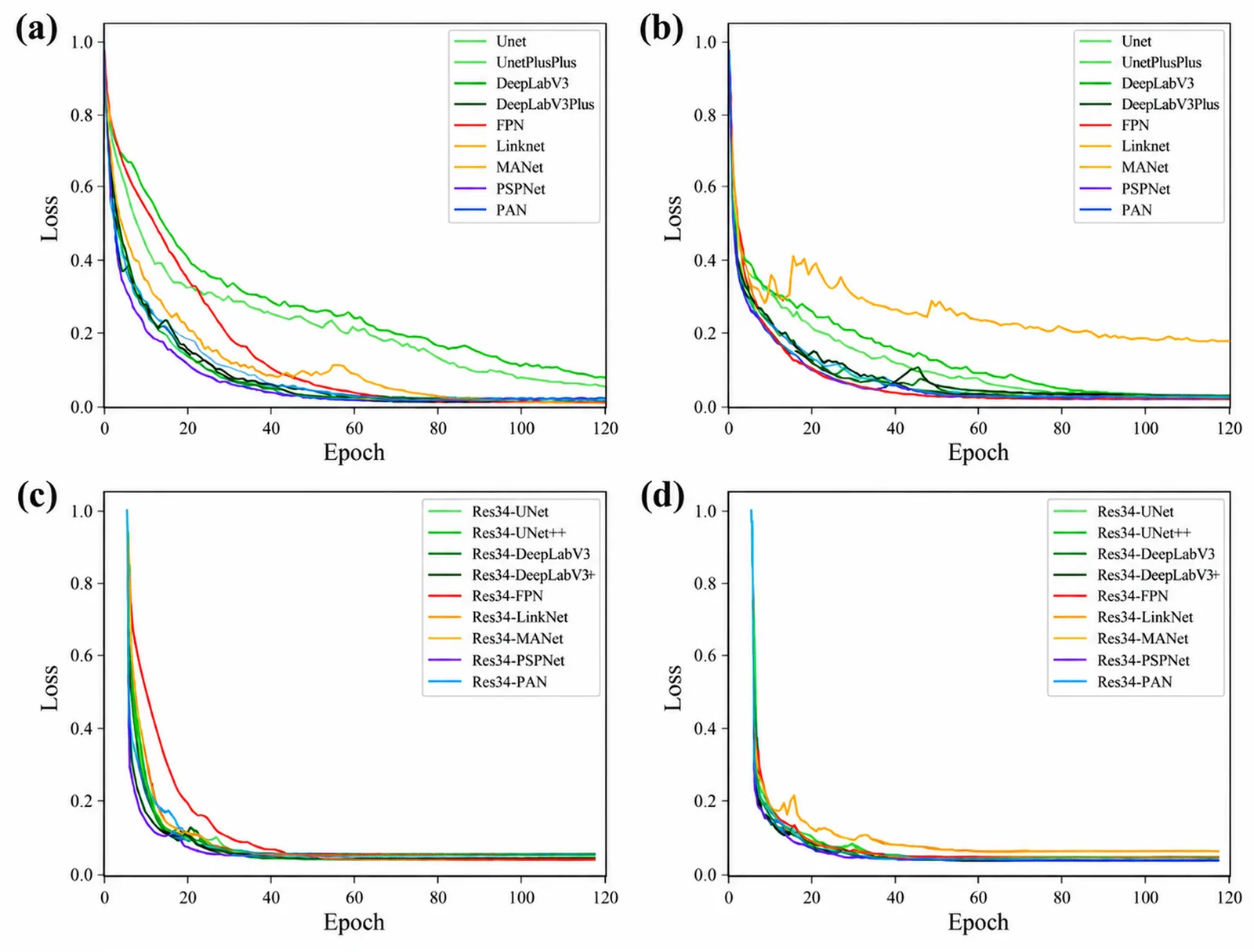
Loss curves during model training on the DDTI and TN3K datasets. The figure shows the variation of the loss function over training epochs under an adaptive learning-rate strategy. Panels (a) and (c) correspond to the DDTI dataset, and panels (b) and (d) correspond to the TN3K dataset. With ReduceLROnPlateau-based learning-rate adjustment and ImageNet-pretrained ResNet34 initialization, the optimized models converge faster and exhibit more stable loss fluctuations compared with the baseline setting.

### Performance of nine classical models on the DDTI and TN3K datasets

Table 2 and Table 3 present the segmentation results of nine classic models on the TN3K and DDTI datasets, respectively. Overall, the models perform better on the TN3K dataset than on the DDTI dataset, suggesting that their stability is compromised when handling ultrasound images with small sample sizes and significant distribution variations. On the TN3K dataset, models like DeepLabV3 and U-Net show similar performance, whereas MANet exhibits weaker results. On the DDTI dataset, performance disparities among the models become more pronounced. The Intersection over Union (IoU) for each model generally falls below 0.65, with FPN, PSPNet, and PAN showing particularly low IoU scores, failing to meet the high-precision demands of medical image segmentation. In thyroid nodule ultrasound images, lesions often feature blurred boundaries, large scale variations, and significant noise, which exacerbate differences in multi-scale context fusion and boundary representation across various structural designs. Some models, such as LinkNet, though low in computational cost, struggle with detail recovery in the decoder. Models with attention-enhancement structures, like MANet, are prone to errors from pseudo-textures and high noise due to their sensitivity to noise and domain differences. Models employing pyramid context aggregation structures, such as PAN, may introduce excessive smoothing in small-target tasks, leading to blurred lesion boundaries.

**Table 2:** Performance metrics of nine classical segmentation models on the TN3K dataset. The performance of nine classical models is reported using IoU, F1-score, accuracy (Acc), specificity (Spec), and precision (Pre). Higher values indicate better segmentation performance.

Models	IoU	F1-score	Acc	Spec	Pre
U-Net	0.7241 ± 0.1691	0.8400 ± 0.1268	0.9477 ± 0.0088	0.9716 ± 0.0214	0.8528 ± 0.0960
U-Net++	0.7096 ± 0.1829	0.8301 ± 0.1451	0.9444 ± 0.0061	0.9694 ± 0.0298	0.8416 ± 0.1127
DeepLabV3	0.7253 ± 0.1770	0.8408 ± 0.1368	0.9479 ± 0.0062	0.9715 ± 0.0266	0.8524 ± 0.0833
DeepLabV3+	0.7074 ± 0.1972	0.8286 ± 0.1647	0.9436 ± 0.0025	0.9677 ± 0.0269	0.8350 ± 0.1083
FPN	0.7008 ± 0.1840	0.8241 ± 0.1443	0.9414 ± 0.0002	0.9640 ± 0.0173	0.8204 ± 0.0842
LinkNet	0.7127 ± 0.1726	0.8323 ± 0.1310	0.9454 ± 0.0061	0.9711 ± 0.0167	0.8488 ± 0.0890
MANet	0.6296 ± 0.1700	0.7727 ± 0.1367	0.9231 ± 0.0023	0.9500 ± 0.0441	0.7580 ± 0.1071
PSPNet	0.6956 ± 0.1575	0.8205 ± 0.1096	0.9418 ± 0.0127	0.9694 ± 0.0193	0.8390 ± 0.1004
PAN	0.7136 ± 0.1938	0.8329 ± 0.1590	0.9463 ± 0.0021	0.9739 ± 0.0212	0.8602 ± 0.0861

**Table 3:** Performance metrics of nine classical segmentation models on the DDTI dataset. The performance of nine classical models is reported using IoU, F1-score, accuracy (Acc), specificity (Spec), and precision (Pre). Higher values indicate better segmentation performance.

Models	IoU	F1-score	Acc	Spec	Pre
U-Net	0.6325 ± 0.1552	0.7749 ± 0.1170	0.9561 ± 0.0296	0.9690 ± 0.0083	0.7281 ± 0.1188
U-Net++	0.6058 ± 0.1525	0.7545 ± 0.1140	0.9539 ± 0.0281	0.9717 ± 0.0090	0.7338 ± 0.1224
DeepLabV3	0.6402 ± 0.1382	0.7806 ± 0.0904	0.9593 ± 0.0341	0.9760 ± 0.0062	0.7682 ± 0.1157
DeepLabV3+	0.6108 ± 0.1576	0.7584 ± 0.1058	0.9530 ± 0.0303	0.9674 ± 0.0085	0.7134 ± 0.1325
FPN	0.5768 ± 0.1430	0.7316 ± 0.1019	0.9476 ± 0.0287	0.9641 ± 0.0128	0.6865 ± 0.1382
LinkNet	0.6288 ± 0.1419	0.7721 ± 0.0915	0.9564 ± 0.0281	0.9710 ± 0.0041	0.7371 ± 0.1174
MANet	0.6346 ± 0.1617	0.7765 ± 0.1131	0.9598 ± 0.0270	0.9792 ± 0.0010	0.7869 ± 0.1229
PSPNet	0.5929 ± 0.1278	0.7444 ± 0.0874	0.9513 ± 0.0300	0.9686 ± 0.0052	0.7131 ± 0.1214
PAN	0.5909 ± 0.1600	0.7429 ± 0.1258	0.9501 ± 0.0285	0.9661 ± 0.0020	0.7006 ± 0.1426

### Performance of the nine improved models on the DDTI and TN3K datasets

Table 4 and Table 5 display the segmentation performance of nine enhanced models using ResNet34 as the encoder on the TN3K and DDTI datasets. Compared to the classic model stage, the IoU and F1-score metrics for each improved model on both datasets have significantly increased, and the performance disparities among the models have narrowed. This suggests that consistently using the ResNet34 encoder effectively boosts feature expression capabilities. On the TN3K dataset, Res34-PAN achieves the best performance in terms of IoU and F1-score, with models like Res34-DeepLabV3+ and Res34-FPN performing similarly. On the DDTI dataset, Res34-PAN continues to demonstrate high segmentation accuracy, while Res34-FPN and Res34-DeepLabV3 + also exhibit strong competitiveness. In contrast, Res34-MANet’s segmentation performance on both datasets remains relatively low, though it shows some improvement over its classic model counterpart. Overall, the introduction of the ResNet34 encoder stabilizes model performance across different datasets, laying a solid foundation for further optimization.

**Table 4:** Performance metrics of nine improved models on the TN3K dataset. The improved models are constructed by replacing the encoder of each classical model with ResNet34 while keeping the original decoder. Results are reported in IoU, F1-score, Acc, Spec, and Pre.

Models	IoU	F1-score	Acc	Spec	Pre
Res34-UNet	0.8077 ± 0.0768	0.8936 ± 0.0196	0.9664 ± 0.0253	0.9795 ± 0.0018	0.8907 ± 0.0496
Res34-UNet++	0.8040 ± 0.0600	0.8913 ± 0.0078	0.9658 ± 0.0225	0.9793 ± 0.0055	0.8898 ± 0.0514
Res34-DeepLabV3	0.8229 ± 0.0711	0.9029 ± 0.0114	0.9694 ± 0.0237	0.9819 ± 0.0064	0.9029 ± 0.0503
Res34-DeepLabV3+	0.8242 ± 0.0800	0.9036 ± 0.0248	0.9697 ± 0.0255	0.9822 ± 0.0019	0.9043 ± 0.0426
Res34-FPN	0.8215 ± 0.0786	0.9020 ± 0.0233	0.9693 ± 0.0219	0.9822 ± 0.0004	0.9043 ± 0.0289
Res34-LinkNet	0.7895 ± 0.0824	0.8824 ± 0.0296	0.9625 ± 0.0252	0.9752 ± 0.0012	0.8707 ± 0.0240
Res34-MANet	0.6804 ± 0.0960	0.8098 ± 0.0426	0.9393 ± 0.0254	0.9614 ± 0.0060	0.7988 ± 0.0047
Res34-PSPNet	0.8033 ± 0.0743	0.8909 ± 0.0132	0.9657 ± 0.0226	0.9796 ± 0.0001	0.8907 ± 0.0255
Res34-PAN	0.8264 ± 0.0672	0.9049 ± 0.0086	0.9701 ± 0.0248	0.9826 ± 0.0019	0.9064 ± 0.0509

**Table 5:** Performance metrics of nine improved models on the DDTI dataset. The improved models are constructed by replacing the encoder of each classical model with ResNet34 while keeping the original decoder. Results are reported in IoU, F1-score, Acc, Spec, and Pre.

Models	IoU	F1-score	Acc	Spec	Pre
Res34-UNet	0.7861 ± 0.1152	0.8803 ± 0.0608	0.9754 ± 0.0026	0.9818 ± 0.0039	0.8462 ± 0.0218
Res34-UNet++	0.7992 ± 0.1132	0.8884 ± 0.0613	0.9775 ± 0.0093	0.9851 ± 0.0028	0.8696 ± 0.0229
Res34-DeepLabV3	0.7934 ± 0.0957	0.8848 ± 0.0472	0.9766 ± 0.0145	0.9838 ± 0.0062	0.8602 ± 0.0164
Res34-DeepLabV3+	0.8152 ± 0.1025	0.8982 ± 0.0374	0.9796 ± 0.0018	0.9870 ± 0.0037	0.8846 ± 0.0077
Res34-FPN	0.8210 ± 0.0928	0.9017 ± 0.0243	0.9803 ± 0.0045	0.9875 ± 0.0056	0.8891 ± 0.0340
Res34-LinkNet	0.7501 ± 0.1109	0.8572 ± 0.0634	0.9698 ± 0.0032	0.9753 ± 0.0015	0.8030 ± 0.0319
Res34-MANet	0.6398 ± 0.0761	0.7803 ± 0.0087	0.9537 ± 0.0155	0.9667 ± 0.0083	0.7329 ± 0.0215
Res34-PSPNet	0.8011 ± 0.1265	0.8896 ± 0.0742	0.9779 ± 0.0059	0.9863 ± 0.0027	0.8780 ± 0.0499
Res34-PAN	0.8434 ± 0.1004	0.9151 ± 0.0359	0.9832 ± 0.0092	0.9905 ± 0.0079	0.9136 ± 0.0068

### Performance of the optimized model using ImageNet weights on the DDTI and TN3K datasets

Building on the improved model that employs ResNet34 as a unified encoder, this paper addresses the challenge of insufficient feature learning in small-sample scenarios by introducing pre-trained weights from ResNet34 on the ImageNet dataset. This initialization strategy aims to enhance the model’s feature transfer capability and improve segmentation performance.

Table 6 and Table 7 present the segmentation performance of optimized models using ImageNet pre-trained weights on the TN3K and DDTI datasets. These tables demonstrate that adopting the ResNet34 encoder with pre-trained weights significantly enhances key indicators like IoU and F1-score across nine models on both datasets. The overall performance surpasses that of models without pre-trained weights. On the TN3K dataset, Res34-PAN achieves the highest IoU and F1-score, indicating superior performance, while models such as Res34-FPN, Res34-DeepLabV3, and Res34-DeepLabV3 + show similar results with minor differences. On the DDTI dataset, Res34-PAN also leads in IoU and F1-score, maintaining stable performance, and the other models exhibit a more concentrated performance distribution. Although Res34-MANet’s segmentation performance is relatively lower compared to others, its IoU and F1-score have improved significantly, narrowing the performance gap. Overall, incorporating ImageNet pre-trained weights enhances the models’ feature transfer capabilities, resulting in higher accuracy and greater stability in small-sample thyroid nodule segmentation tasks.

**Table 6:** Performance metrics of nine optimized models on the TN3K dataset. The optimized models initialize the ResNet34 encoder with ImageNet-pretrained weights. Results are reported in IoU, F1-score, Acc, Spec, and Pre.

Models	IoU	F1-score	Acc	Spec	Pre
Res34-UNet	0.8540 ± 0.0676	0.9212 ± 0.0136	0.9752 ± 0.0067	0.9854 ± 0.0049	0.9216 ± 0.0316
Res34-UNet++	0.8588 ± 0.0588	0.9241 ± 0.0008	0.9762 ± 0.0097	0.9862 ± 0.0006	0.9258 ± 0.0527
Res34-DeepLabV3	0.8635 ± 0.0677	0.9268 ± 0.0139	0.9770 ± 0.0105	0.9864 ± 0.0016	0.9269 ± 0.0410
Res34-DeepLabV3+	0.8617 ± 0.0559	0.9257 ± 0.0003	0.9766 ± 0.0106	0.9862 ± 0.0011	0.9262 ± 0.0473
Res34-FPN	0.8652 ± 0.0759	0.9277 ± 0.0249	0.9773 ± 0.0069	0.9872 ± 0.0085	0.9310 ± 0.0361
Res34-LinkNet	0.8565 ± 0.0630	0.9227 ± 0.0067	0.9757 ± 0.0093	0.9856 ± 0.0002	0.9230 ± 0.0301
Res34-MANet	0.7794 ± 0.0852	0.8760 ± 0.0251	0.9610 ± 0.0009	0.9770 ± 0.0041	0.8764 ± 0.0215
Res34-PSPNet	0.8536 ± 0.0813	0.9210 ± 0.0269	0.9752 ± 0.0076	0.9857 ± 0.0013	0.9230 ± 0.0216
Res34-PAN	0.8734 ± 0.0627	0.9324 ± 0.0092	0.9788 ± 0.0111	0.9884 ± 0.0051	0.9370 ± 0.0401

**Table 7:** Performance metrics of nine optimized models on the DDTI dataset. The optimized models initialize the ResNet34 encoder with ImageNet-pretrained weights. Results are reported in IoU, F1-score, Acc, Spec, and Pre.

Models	IoU	F1-score	Acc	Spec	Pre
Res34-UNet	0.8386 ± 0.0428	0.9122 ± 0.0258	0.9823 ± 0.0097	0.9879 ± 0.0046	0.8938 ± 0.0464
Res34-UNet++	0.8424 ± 0.0589	0.9144 ± 0.0084	0.9827 ± 0.0107	0.9880 ± 0.0075	0.8949 ± 0.0387
Res34-DeepLabV3	0.8276 ± 0.0427	0.9057 ± 0.0265	0.9807 ± 0.0060	0.9852 ± 0.0100	0.8739 ± 0.0778
Res34-DeepLabV3+	0.8325 ± 0.0516	0.9086 ± 0.0176	0.9815 ± 0.0072	0.9867 ± 0.0035	0.8848 ± 0.0462
Res34-FPN	0.8388 ± 0.0791	0.9123 ± 0.0213	0.9824 ± 0.0086	0.9882 ± 0.0035	0.8958 ± 0.0097
Res34-LinkNet	0.8349 ± 0.0731	0.9100 ± 0.0023	0.9815 ± 0.0054	0.9852 ± 0.0005	0.8753 ± 0.0260
Res34-MANet	0.8017 ± 0.0604	0.8899 ± 0.0106	0.9771 ± 0.0048	0.9815 ± 0.0001	0.8471 ± 0.0427
Res34-PSPNet	0.8132 ± 0.0906	0.8970 ± 0.0207	0.9792 ± 0.0071	0.9860 ± 0.0007	0.9216 ± 0.0120
Res34-PAN	0.8778 ± 0.0415	0.9349 ± 0.0268	0.9872 ± 0.0112	0.9928 ± 0.0054	0.9342 ± 0.0570

### Performance improvement

The performance enhancements of the improved model, as compared to the classical model, are detailed in Table 8 and Table 9.

**Table 8:** Performance gains of improved and optimized models on the TN3K dataset. The table reports the performance improvements of the ResNet34-based improved models and the ImageNet-pretrained optimized models relative to their corresponding classical baselines on TN3K, measured by IoU, F1-score, Acc, Spec, and Pre. Positive values indicate improvements.

Models	ImageNet	IoU	F1-score	Acc	Spec	Pre
U-Net	FALSE	+0.1155	+0.0638	+0.0197	+0.0081	+0.0444
	TRUE	+0.1794	+0.0967	+0.0290	+0.0142	+0.0807
U-Net++	FALSE	+0.1330	+0.0737	+0.0227	+0.0102	+0.0573
	TRUE	+0.2103	+0.1132	+0.0337	+0.0173	+0.1000
DeepLabV3	FALSE	+0.1346	+0.0739	+0.0227	+0.0107	+0.0592
	TRUE	+0.1905	+0.1023	+0.0307	+0.0153	+0.0874
DeepLabV3+	FALSE	+0.1651	+0.0905	+0.0277	+0.0150	+0.0830
	TRUE	+0.2181	+0.1172	+0.0350	+0.0191	+0.1092
FPN	FALSE	+0.1722	+0.0945	+0.0296	+0.0189	+0.1023
	TRUE	+0.2346	+0.1257	+0.0381	+0.0241	+0.1348
LinkNet	FALSE	+0.1078	+0.0602	+0.0181	+0.0042	+0.0258
	TRUE	+0.2018	+0.1086	+0.0320	+0.0149	+0.0874
MANet	FALSE	+0.0807	+0.0480	+0.0175	+0.0120	+0.0538
	TRUE	+0.2379	+0.1337	+0.0411	+0.0284	+0.1562
PSPNet	FALSE	+0.1548	+0.0858	+0.0254	+0.0105	+0.0616
	TRUE	+0.2271	+0.1225	+0.0355	+0.0168	+0.1001
PAN	FALSE	+0.1581	+0.0864	+0.0252	+0.0089	+0.0537
	TRUE	+0.2239	+0.1195	+0.0343	+0.0149	+0.0893

**Table 9:** Performance gains of improved and optimized models on the DDTI dataset. The table reports the performance improvements of the ResNet34-based improved models and the ImageNet-pretrained optimized models relative to their corresponding classical baselines on DDTI, measured by IoU, F1-score, Acc, Spec, and Pre. Positive values indicate improvements.

Models	ImageNet	IoU	F1-score	Acc	Spec	Pre
U-Net	FALSE	+0.2428	+0.1360	+0.0202	+0.0132	+0.1622
	TRUE	+0.3258	+0.1772	+0.0274	+0.0195	+0.2276
U-Net++	FALSE	+0.3192	+0.1775	+0.0247	+0.0138	+0.1851
	TRUE	+0.3906	+0.2119	+0.0302	+0.0168	+0.2195
DeepLabV3	FALSE	+0.2393	+0.1335	+0.0180	+0.0080	+0.1198
	TRUE	+0.2927	+0.1603	+0.0223	+0.0094	+0.1376
DeepLabV3+	FALSE	+0.3346	+0.1843	+0.0279	+0.0203	+0.2400
	TRUE	+0.3630	+0.1980	+0.0299	+0.0200	+0.2403
FPN	FALSE	+0.4234	+0.2325	+0.0345	+0.0243	+0.2951
	TRUE	+0.4542	+0.2470	+0.0367	+0.0250	+0.3049
LinkNet	FALSE	+0.1929	+0.1102	+0.0140	+0.0044	+0.0894
	TRUE	+0.3278	+0.1786	+0.0262	+0.0146	+0.1875
MANet	FALSE	+0.0082	+0.0049	−0.0064	−0.0128	−0.0686
	TRUE	+0.2633	+0.1460	+0.0180	+0.0023	+0.0765
PSPNet	FALSE	+0.3512	+0.1951	+0.0280	+0.0183	+0.2312
	TRUE	+0.3716	+0.2050	+0.0293	+0.0180	+0.2924
PAN	FALSE	+0.4273	+0.2318	+0.0348	+0.0253	+0.3040
	TRUE	+0.4855	+0.2584	+0.0390	+0.0276	+0.2529

Experimental data indicate that in the TN3K and DDTI datasets, the nine enhanced models with ImageNet pre-trained weights generally show significant improvement. By incorporating ResNet34 into the encoder of the classic model and utilizing pre-trained weights, the model’s feature extraction capability is substantially enhanced. Data analysis reveals that on the TN3K dataset, MANet and FPN exhibit the most notable performance improvements, with their IoU increasing by 0.2379 and 0.2346, respectively. In the DDTI dataset, the PAN and FPN models demonstrate the greatest optimization, with IoU improvements of 0.4855 and 0.4542, respectively. This suggests that the new encoder significantly enhances model optimization, effectively addressing the original model’s shortcomings in medical image feature extraction and lesion recognition.

To more clearly and intuitively present the models’ performance, this experiment visualizes the indicators of nine classic models alongside the optimized model, as shown in Fig 7 and Fig 8.

**Fig 7:**
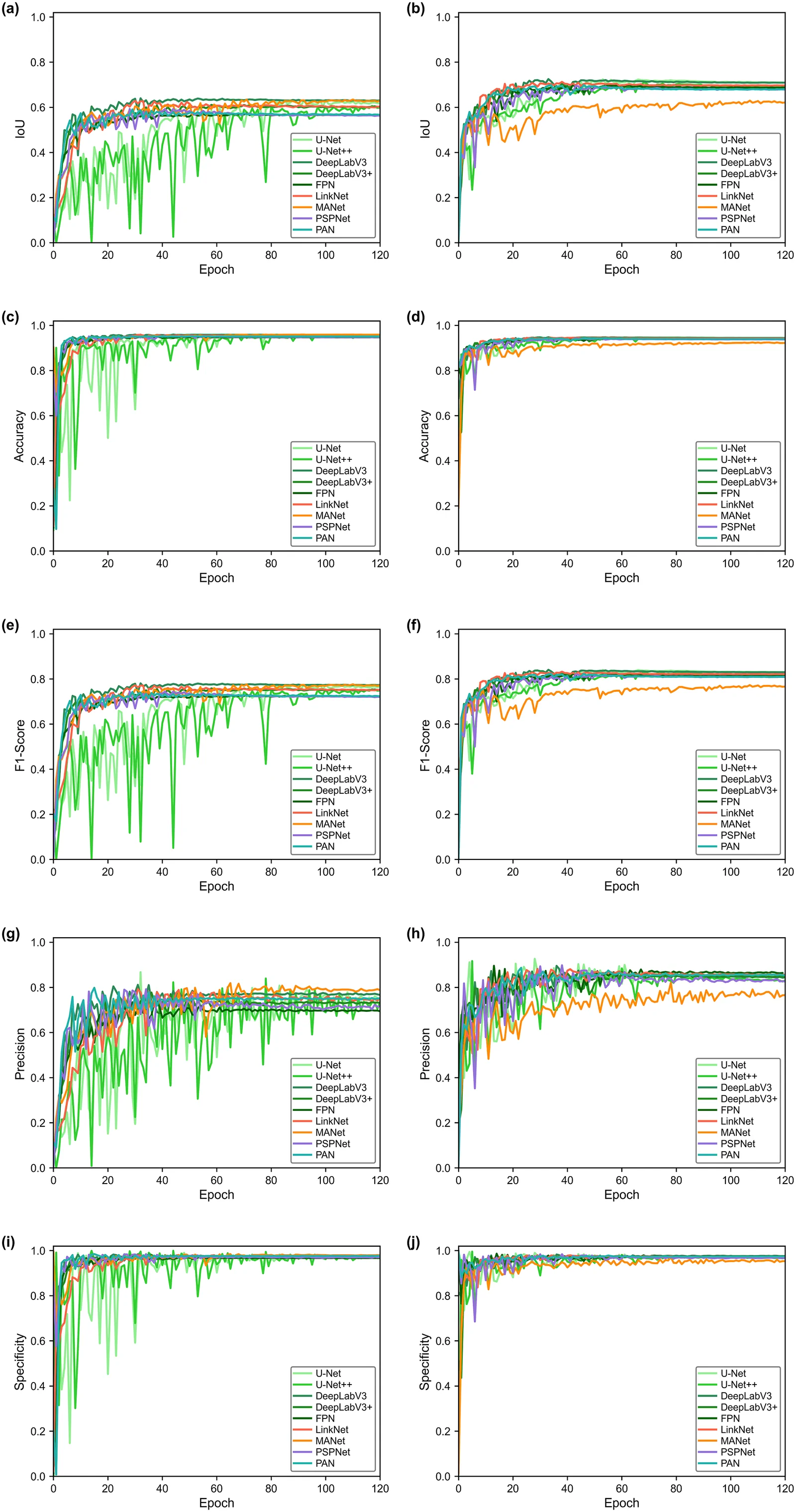
Segmentation performance of nine classical models on the DDTI and TN3K datasets. Panels (a), (c), (e), (g), and (i) report the performance of the nine classical segmentation networks on the DDTI dataset, while panels (b), (d), (f), (h), and (j) report the corresponding performance on the TN3K dataset. Five evaluation metrics are presented: IoU, F1-score, accuracy, specificity, and precision.

**Fig 8:**
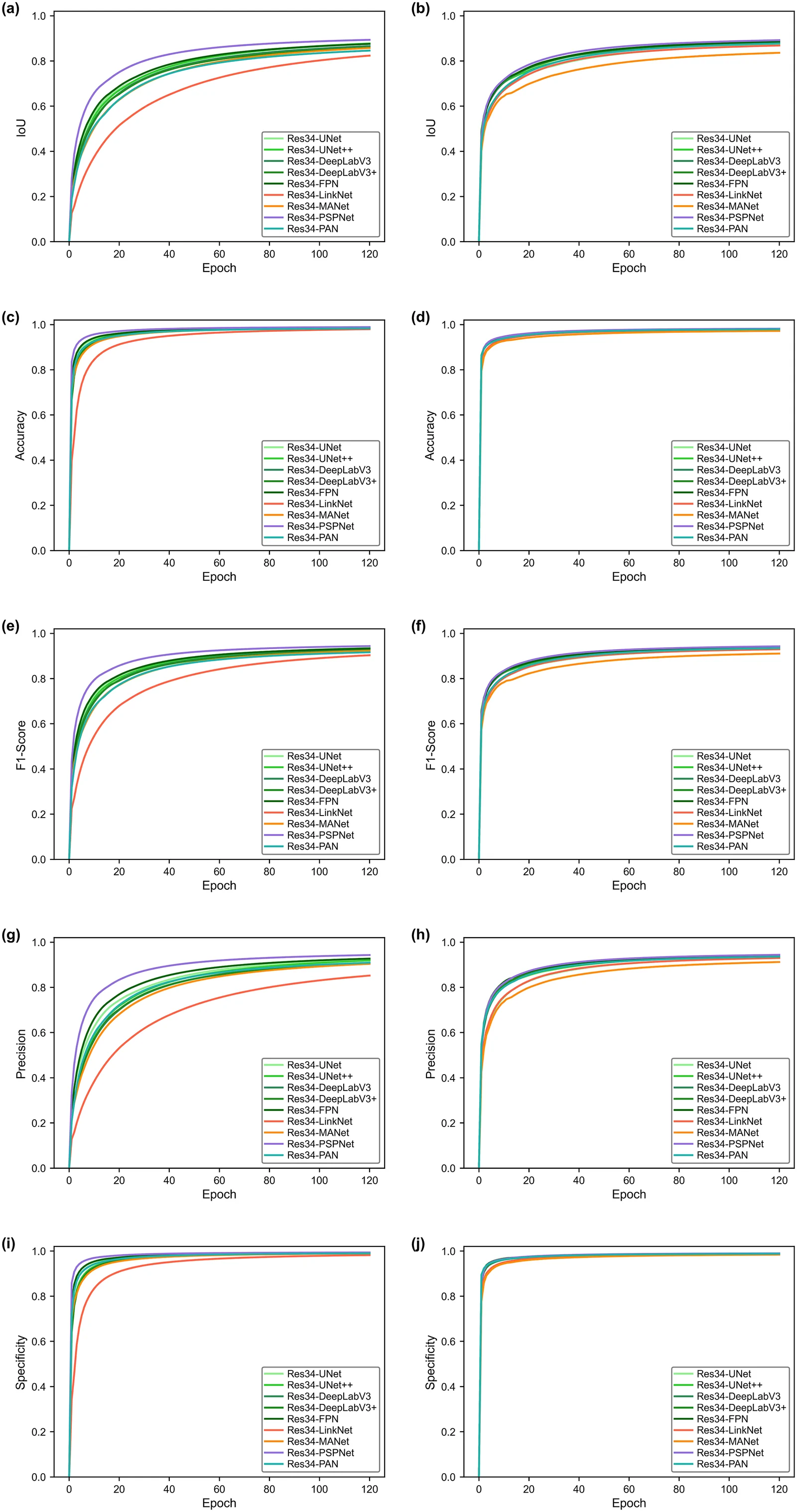
Segmentation performance of the optimized ResNet34-based models on the DDTI and TN3K datasets. The optimized models are constructed by replacing the encoder of each classical network with ResNet34 and initializing the backbone with ImageNet-pretrained weights. Panels (a), (c), (e), (g), and (i) show the results on the DDTI dataset, and panels (b), (d), (f), (h), and (j) show the results on the TN3K dataset. Five evaluation metrics are reported: IoU, F1-score, accuracy, specificity, and precision.

Analyzing the IoU and Precision curves over training iterations reveals that segmentation performance generally improves with more epochs. However, comparing the classic and improved models shows significant differences in training stability and convergence characteristics. Early in training, the classic model’s IoU and Precision curves fluctuate significantly, indicating unstable parameter updates and slow convergence. This instability may stem from the limited number of medical image samples and challenges in random initialization for feature learning. In contrast, the improved model’s curves are smoother, exhibiting a rapid rise and eventual stabilization, suggesting that pre-trained feature priors effectively accelerate convergence and enhance training stability. Regarding the Precision metric, the classic model often shows a rise followed by a decline, indicating unstable discrimination for challenging samples, whereas the improved model exhibits significantly reduced oscillation. Overall, pre-trained initialization enhances both convergence speed and segmentation accuracy. Notably, Res34-PAN consistently maintains high performance across all metrics, further validating its superiority and generalization capability in thyroid nodule segmentation tasks.

This study visually presents the segmentation outcomes of the models. Specifically, three representative images were selected from each of the TN3K and DDTI datasets to illustrate cases with relatively good and poor segmentation performance. These images were segmented using the classical models and the corresponding enhanced ResNet34-based models proposed in this study. The resulting segmentation outcomes are shown in Fig 9 and Fig 10.

**Fig 9:**
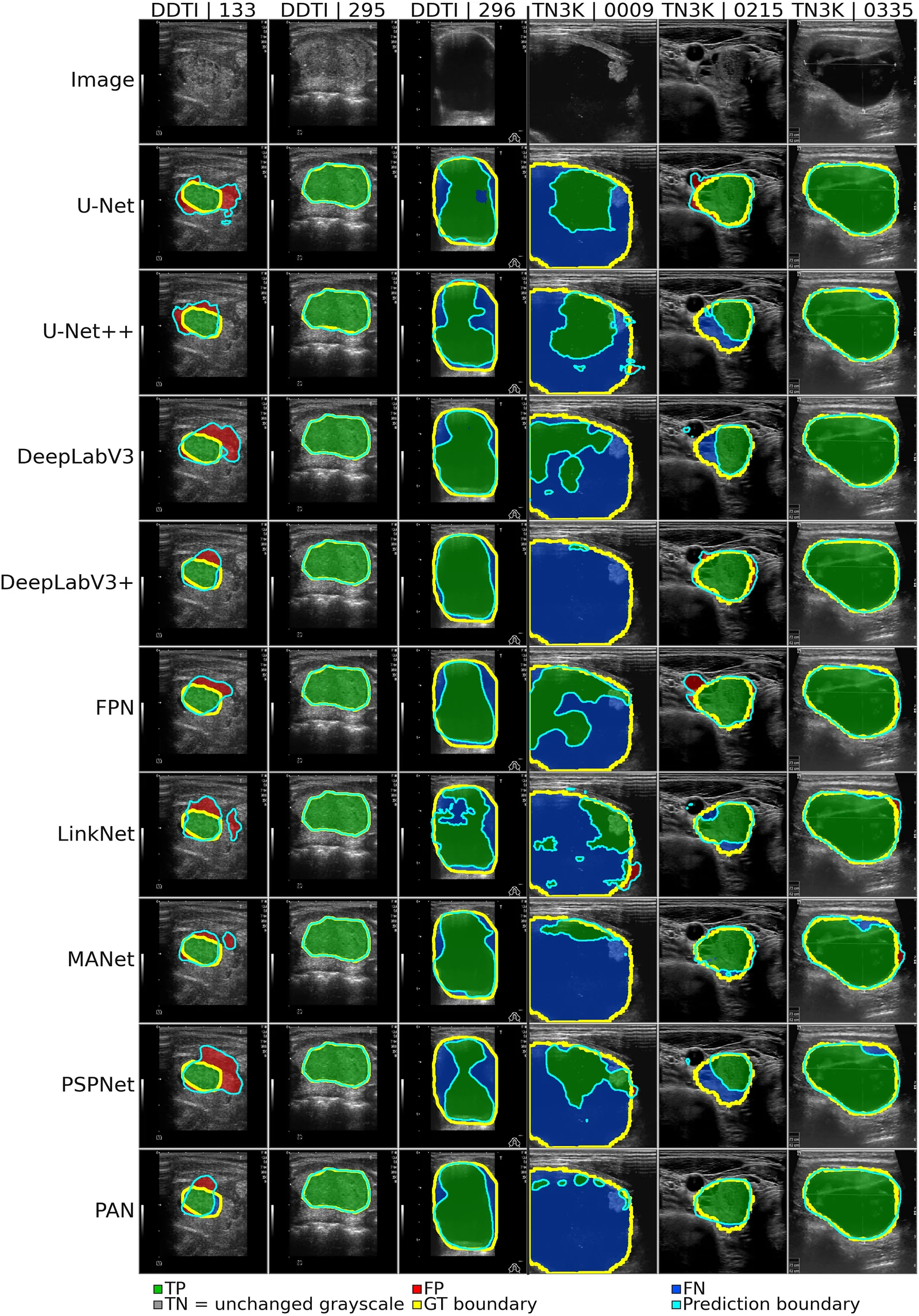
Qualitative segmentation results of nine classical models on the DDTI and TN3K datasets. Representative samples with both good and poor segmentation performance are selected from the two datasets for visualization. The first three cases are from the DDTI dataset, while the last three cases are from the TN3K dataset. For each case, the first row shows the original ultrasound image, and the second to tenth rows show the segmentation results produced by U-Net, U-Net++, DeepLabV3, DeepLabV3 + , FPN, LinkNet, MANet, PSPNet, and PAN, respectively. The segmentation results are visualized using color-coded overlays, where true-positive (TP) regions are shown in green, false-positive (FP) regions in red, and false-negative (FN) regions in blue, while true-negative (TN) regions retain the original grayscale background. In addition, the ground-truth (GT) boundary is outlined in yellow and the predicted boundary in cyan, enabling simultaneous visualization of the reference annotation and model prediction for intuitive comparison of segmentation accuracy and boundary agreement.

**Fig 10:**
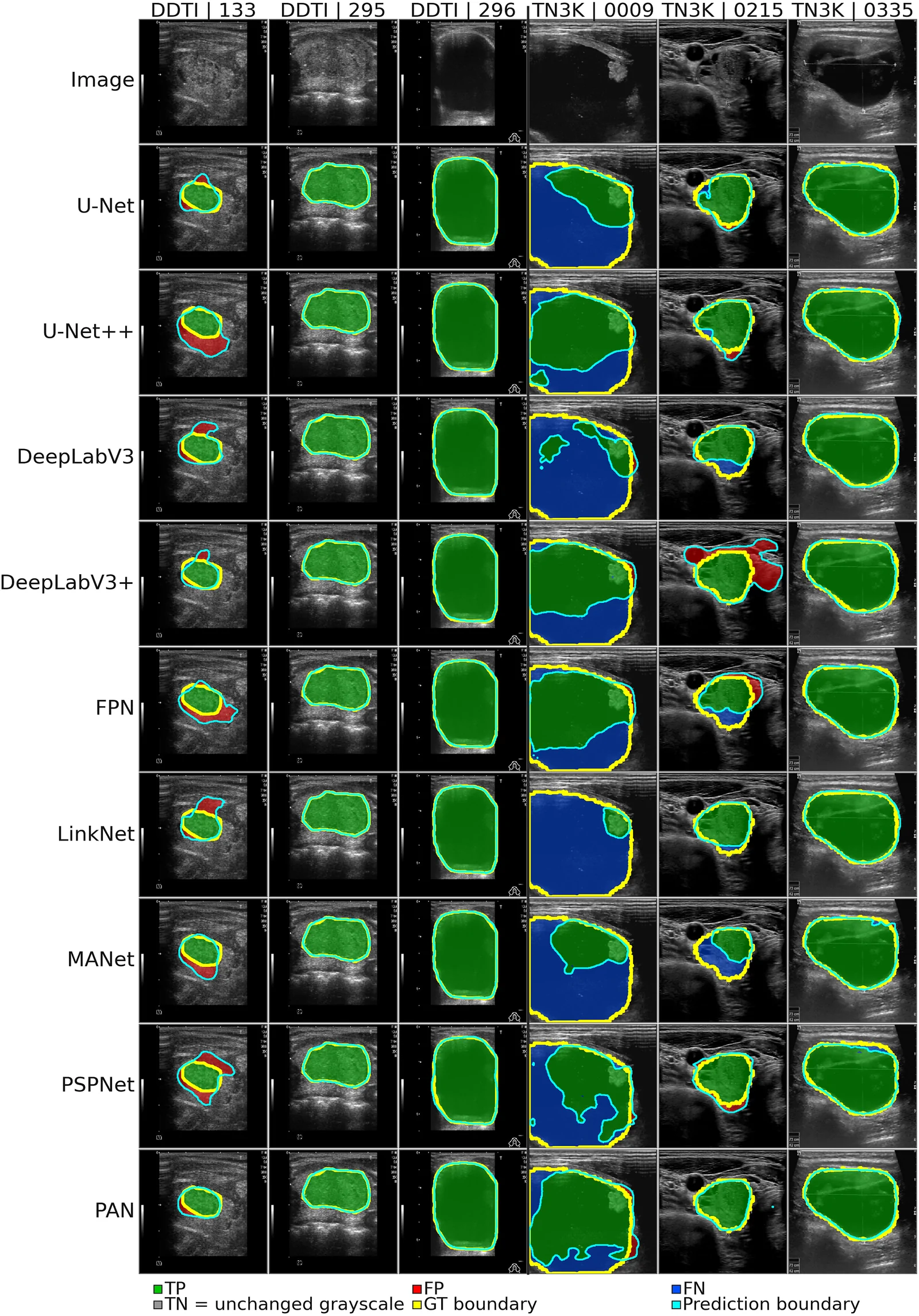
Qualitative segmentation results of nine optimized ResNet34-based models on the DDTI and TN3K datasets. The same representative samples as those shown in Fig 9 are used for visualization, including cases with both good and poor segmentation performance. The first three cases are from the DDTI dataset, while the last three cases are from the TN3K dataset. For each case, the first row shows the original ultrasound image, and the second to tenth rows show the segmentation results produced by Res34-UNet, Res34-UNet++, Res34-DeepLabV3, Res34-DeepLabV3 + , Res34-FPN, Res34-LinkNet, Res34-MANet, Res34-PSPNet, and Res34-PAN, respectively. The segmentation results are visualized using color-coded overlays, where true-positive (TP) regions are shown in green, false-positive (FP) regions in red, and false-negative (FN) regions in blue, while true-negative (TN) regions retain the original grayscale background. In addition, the ground-truth (GT) boundary is outlined in yellow and the predicted boundary in cyan, enabling simultaneous visualization of the reference annotation and model prediction for intuitive comparison of segmentation accuracy and boundary agreement.

Based on experimental findings across various models, it was observed that in the task of segmenting thyroid nodules, basic models exhibited a higher tendency for erroneous segmentation compared to optimized models. Specifically, misclassification of the background as the target region led to subpar segmentation outcomes. While optimized models still displayed some segmentation errors relative to the gold standard, they were able to accurately indicate the approximate location and extent of the nodules. Among the 18 models examined, Res34-PAN demonstrated the most favorable segmentation results, closely approximating the ground truth and outperforming alternative algorithms. Analysis of the segmentation models’ boundary contours revealed that Res34-PAN exhibited sharper and smoother boundaries, underscoring the efficacy of model optimization in enhancing segmentation performance. Across diverse datasets, Res34-PAN consistently delivered strong segmentation performance and exhibited robust model performance.

### Ablation experiment

To further validate the encoder replacement strategy proposed in this paper and examine how various backbone network structures and pre-training initialization methods affect the model’s segmentation performance, we conducted a systematic ablation experiment using the TN3K dataset with the PAN model. In this experiment, we selected commonly used lightweight networks like MobileNetV2 and EfficientNet-B0, along with residual networks of varying depths, including ResNet18, ResNet34, ResNet50, ResNet101, and ResNet152, as potential encoder structures. We compared the models’ performance under two conditions: random initialization (Pretrained = False) and loading pre-trained ImageNet weights (Pretrained = True). The experimental results are presented in Table 10.

**Table 10:** Ablation results of PAN on the TN3K dataset. This table compares different encoder backbones used in PAN and evaluates the effect of ImageNet-pretrained initialization. Results are reported in IoU, F1-score, Acc, Spec, and Pre.

Encoder	Pretrained	IoU	Duration(min)	Params(M)	VRAM(GB)
Efficientnet	FALSE	0.7145	16.22	4.10	9.12
	TRUE	0.7702	19.21	4.10	9.12
Mobilenet	FALSE	0.6514	12.88	2.42	9.12
	TRUE	0.7473	12.81	2.42	9.12
Resnet101	FALSE	0.7184	21.48	43.25	6.75
	TRUE	0.7389	21.75	43.25	6.75
Resnet152	FALSE	0.7309	27.45	58.90	9.12
	TRUE	0.7425	27.06	58.90	9.12
Resnet18	FALSE	0.7319	9.61	11.37	1.42
	TRUE	0.7505	9.84	11.37	1.42
Resnet34	FALSE	0.7315	12.35	21.48	2.03
	TRUE	0.8778	12.51	21.48	2.03
Resnet50	FALSE	0.7220	16.46	24.26	4.78
	TRUE	0.7462	16.62	24.26	4.78

The experimental results demonstrate that varying encoder structures significantly affect the segmentation performance of the PAN model. When pre-trained weights are not used, the Intersection over Union (IoU) values for each encoder range from 0.65 to 0.73. MobileNet, with only 2.42M parameters and the shortest training time, achieves an IoU of just 0.6514, indicating that lightweight networks still lack sufficient feature expression capability for complex medical imaging tasks. In contrast, the ResNet series exhibits superior performance overall. ResNet18 and ResNet34 achieve IoU values of 0.7319 and 0.7315, respectively, suggesting that the residual structure offers more stable feature extraction capabilities for small-sample ultrasound data.

Introducing ImageNet pre-trained weights enhances model accuracy, confirming the efficacy of pre-training strategies in small-sample medical image segmentation. For instance, EfficientNet’s IoU improves from 0.7145 to 0.7702, and MobileNet’s from 0.6514 to 0.7473, demonstrating that pre-trained features can mitigate training challenges due to limited data. Conversely, ResNet101 and ResNet152, with their large parameter scales, require more training time and achieve a maximum IoU of only 0.7425, suggesting that overly deep networks may struggle with the current data scale. ResNet34, however, achieves optimal results with pre-trained weights, significantly boosting its IoU from 0.7315 to 0.8778. It also maintains a training time of just 12.51 minutes and a memory usage of 2.03 GB, striking a balance between accuracy and computational efficiency. In summary, the choice of encoder and pre-training initialization significantly impacts PAN performance, with ResNet34 offering the best overall performance. Consequently, this study selects ResNet34 as the unified encoder for subsequent model improvement experiments.

### Robustness test

To further evaluate the generalization capability and robustness of the proposed model under different data distributions, a cross-dataset evaluation was conducted using Res34-PAN, which achieved the best segmentation performance on the TN3K dataset. Specifically, Res34-PAN was first trained on the TN3K dataset, and the model weights corresponding to the best validation performance were retained. The trained model was then directly applied to the entire DDTI dataset for segmentation without any additional training, fine-tuning, or parameter updating. Therefore, the DDTI dataset served as an independent external test dataset in this experiment to evaluate the transferability and generalization capability of the model across different data domains.

Considering the potential differences between the TN3K and DDTI datasets in terms of image acquisition devices, imaging conditions, grayscale distributions, noise characteristics, and nodule morphology, this cross-dataset evaluation provides a more stringent assessment of model stability under distribution shifts. Unlike the previous evaluation based on a limited number of randomly selected images, the revised experiment performs inference on all images in the DDTI dataset. For each image, five evaluation metrics, including Intersection over Union (IoU), F1-score, Accuracy (Acc), Specificity (Spec), and Precision (Pre), are calculated, thereby providing a sample-level performance distribution over the entire external dataset.

To provide a more comprehensive representation of model performance across individual cases, box plots are used to summarize the distributions of the five evaluation metrics over the entire DDTI dataset, as shown in Fig 11. The boxes represent the interquartile ranges of the corresponding metrics, while the central lines indicate the medians. The mean values, observed minimum and maximum values, and outliers identified according to Tukey’s criterion are also displayed. Compared with reporting the performance of only a limited number of individual samples, this distribution-based analysis provides a more informative assessment of the overall performance, inter-sample variability, and extreme prediction cases of the model.

**Fig 11:**
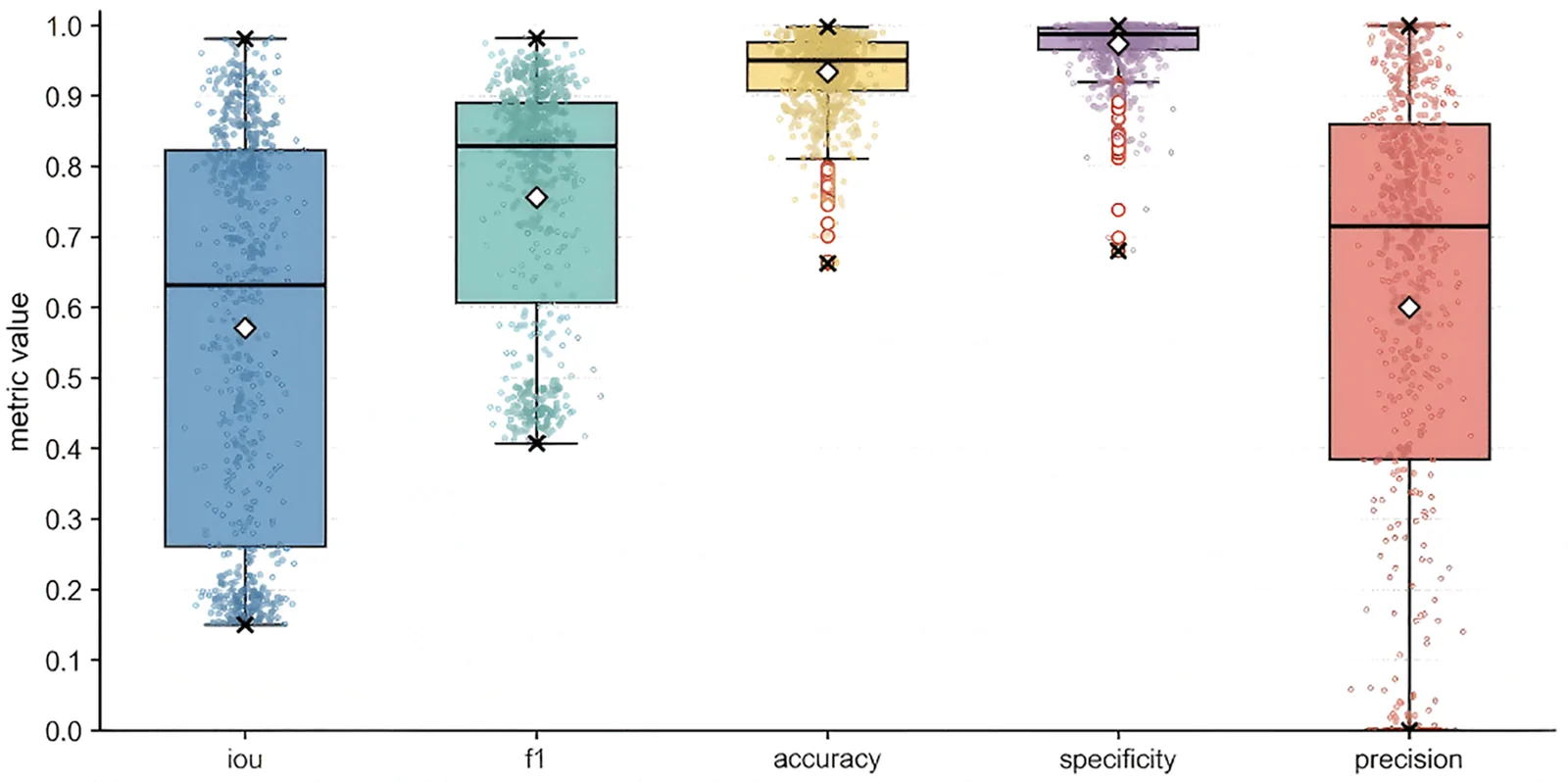
Cross-dataset robustness evaluation of Res34-PAN trained on TN3K and directly tested on the entire DDTI dataset. The distributions of per-image IoU, F1-score, accuracy, specificity, and precision are presented as box plots. The model is evaluated on DDTI using the weights obtained from TN3K without any retraining or fine-tuning. The boxes represent the interquartile ranges, the central lines indicate the medians, diamonds indicate the means, crosses denote the observed minimum and maximum values, and circles represent outliers identified according to Tukey’s criterion.

As shown in Fig 11, Res34-PAN remains capable of segmenting thyroid nodule regions on the external DDTI dataset without retraining or fine-tuning. The distributions of Accuracy and Specificity are relatively concentrated, whereas greater variability can be observed for IoU, F1-score, and Precision across different samples. This suggests that the model maintains relatively stable discrimination of background regions, while its lesion localization and boundary delineation performance may decrease for some challenging ultrasound images with complex nodule morphology, low contrast, blurred boundaries, or substantial noise. The low-value samples and outliers observed in the box plots further indicate that difficult cases remain challenging under cross-dataset distribution shifts.

Overall, the experiment demonstrates that the Res34-PAN model trained exclusively on the TN3K dataset can be directly transferred to the DDTI dataset without target-domain fine-tuning while retaining a certain degree of segmentation capability and stability. These findings further support the effectiveness of the ResNet34 encoder and ImageNet-pretrained initialization in enhancing feature transferability, while also indicating that domain differences between ultrasound datasets remain an important factor affecting cross-dataset segmentation performance.

## Conclusions

Public datasets of 2D medical images of thyroid nodules are currently limited. Additionally, variations in annotation quality and dataset scale across these datasets hinder the model’s generalization ability, necessitating performance improvements. During model training, residual networks effectively train deep networks, circumvent gradient vanishing issues, enhance image feature learning, and capture both local and global image information, thereby boosting overall segmentation performance. Common residual networks include ResNet18, ResNet34, ResNet50, and ResNet101, with complexity increasing alongside the number of layers. Experimental comparisons indicate that ResNet34 excels in thyroid nodule segmentation tasks, making it the chosen residual network for this study. Compared to the original basic model, it enhances segmentation performance in accurate recognition, positioning ability, iteration speed, and generalization, offering a new direction for small-sample tasks like thyroid nodule lesion recognition. Furthermore, incorporating ResNet34 weights trained on ImageNet can further enhance model performance, partially offsetting the impact of limited data.

In this experiment, while the Res34-PAN model demonstrates commendable segmentation performance, the next research phase aims to simultaneously enhance the learning of both regional and boundary features, establish their correlation, and achieve more precise segmentation to surpass current performance limits. Future efforts should focus on optimizing the model to boost recognition accuracy. Additionally, the model’s segmentation performance should be evaluated on a broader range of thyroid nodule test sets and diverse disease datasets to improve its generalization capabilities.

## References

[1] Welker MJ, Orlov D. Thyroid nodules. Am Fam Phys. 2003;67(3):559–67. 12588078

[2] Popoveniuc G, Jonklaas J. Thyroid nodules. Med Clin North Am. 2012;96(2):329–49. 10.1016/j.mcna.2012.02.002 PMC3575959 22443979

[3] Hegedüs L. The thyroid nodule. N Engl J Med. 2004;351(17):1764–71. doi: 10.1056/nejmcp031436 15496625

[4] Burman KD, Wartofsky L. Thyroid nodules. N Engl J Med. 2015;373(24):2347–56. 10.1056/NEJMcp1415786 26650154

[5] Poller DN, Stelow EB, Yiangou C. Thyroid FNAC cytology: can we do it better? Cytopathology. 2008;19(1):4–10. doi: 10.1111/j.1365-2303.2007.00492.x 17916090

[6] Zhu Y, Song Y, Xu G, Fan Z, Ren W. Causes of misdiagnoses by thyroid fine-needle aspiration cytology (FNAC): our experience and a systematic review. Diagn Pathol. 2020;15(1):1. doi: 10.1186/s13000-019-0924-z 31900180 PMC6942345

[7] Yeung MJ, Serpell JW. Management of the solitary thyroid nodule. Oncologist. 2008;13(2):105–12. doi: 10.1634/theoncologist.2007-0212 18305054

[8] Bernardi S, Rosolen V, Barbone F, Borgato S, Deandrea M, De Feo P, et al. Clinical outcomes of thermal ablation re-treatment of benign thyroid nodules: A multicenter study from the italian minimally invasive treatments of the thyroid group. Thyroid. 2024;34(3):360–70. doi: 10.1089/thy.2023.0501 38149599

[9] Mehanna H, Sidhu PS, Madani G, Woolley R, Boelaert K, Nankivell P, et al. Evaluation of US Elastography in Thyroid Nodule Diagnosis: The ElaTION Randomized Control Trial. Radiology. 2024;313(1):e240705. doi: 10.1148/radiol.240705 39404634

[10] Giovanella L, Campennì A, Tuncel M. Integrated diagnostics of thyroid nodules. Cancers. 2024;16(2):311. 10.3390/cancers16020311 PMC10814240 38254799

[11] Long J, Shelhamer E, Darrell T. Fully convolutional networks for semantic segmentation. Proceedings of the IEEE conference on computer vision and pattern recognition. 2015. pp. 3431–40. 10.1109/TPAMI.2016.2572683 27244717

[12] LeCun Y, Boser B, Denker JS, Henderson D, Howard RE, Hubbard W, et al. Backpropagation applied to handwritten zip code recognition. Neural Comput. 1989;1(4):541–51. doi: 10.1162/neco.1989.1.4.541

[13] Ronneberger O, Fischer P, Brox T. U-net: Convolutional networks for biomedical image segmentation. Medical image computing and computer-assisted intervention–MICCAI 2015: 18th international conference, Munich, Germany, October 5-9, 2015, proceedings, part III 18. Springer International Publishing; 2015. pp. 234–41.

[14] Zhou Z, Rahman Siddiquee MM, Tajbakhsh N. Unet: A nested u-net architecture for medical image segmentation. In: Deep Learning in Medical Image Analysis and Multimodal Learning for Clinical Decision Support: 4th International Workshop, DLMIA 2018, and 8th International Workshop, ML-CDS 2018, Held in Conjunction with MICCAI 2018, Granada, Spain, September 20, 2018, Proceedings 4. Springer International Publishing; 2018. pp. 3–11. 10.1007/978-3-030-00889-5_1 PMC7329239 32613207

[15] He K, Zhang X, Ren S. Deep residual learning for image recognition. In: Proceedings of the IEEE conference on computer vision and pattern recognition. 2016. pp. 770–8.

[16] Wan Ahmad WSHM, Zaki WMDW, Ahmad Fauzi MF. Lung segmentation on standard and mobile chest radiographs using oriented Gaussian derivatives filter. Biomed Eng Online. 2015;14:20. doi: 10.1186/s12938-015-0014-8 25889188 PMC4355502

[17] Goceri E. Nuclei segmentation using attention aware and adversarial networks. Neurocomputing. 2024;579:127445. doi: 10.1016/j.neucom.2024.127445

[18] Na S, Guo Y, Jiang F, et al. Segment any cell: A SAM-based auto-prompting fine-tuning framework for nuclei segmentation. 2024. 10.1109/TNNLS.2025.3611322 41144418

[19] Rezvani S, Fateh M, Jalali Y, Fateh A. FusionLungNet: Multi-scale fusion convolution with refinement network for lung CT image segmentation. Biomed Signal Process Control. 2025;107:107858. doi: 10.1016/j.bspc.2025.107858

[20] Hasan D, Abdulazeez AM. Lung segmentation from chest X-ray images using Deeplabv3plus-based CNN Model. Indo J Comput Sci. 2024;13(1). doi: 10.33022/ijcs.v13i1.3700

[21] Sulaiman A, Anand V, Gupta S, Rajab A, Alshahrani H, Al Reshan MS, et al. Attention based UNet model for breast cancer segmentation using BUSI dataset. Sci Rep. 2024;14(1):22422. doi: 10.1038/s41598-024-72712-5 39341859 PMC11439015

[22] Samudrala S, Mohan CK. Semantic segmentation of breast cancer images using DenseNet with proposed PSPNet. Multimed Tools Appl. 2023;83(15):46037–63. doi: 10.1007/s11042-023-17411-5

[23] Chen LC. Rethinking atrous convolution for semantic image segmentation. arXiv preprint. 2017. doi: 10.48550/arXiv.1706.05587

[24] Chen L-C, Zhu Y, Papandreou G, Schroff F, Adam H. Encoder-Decoder with Atrous Separable Convolution for Semantic Image Segmentation. Proceedings of the European conference on computer vision (ECCV). 2018. pp. 833–51. doi: 10.1007/978-3-030-01234-2_49

[25] Lin TY, Dollár P, Girshick R. Feature pyramid networks for object detection. In: Proceedings of the IEEE conference on computer vision and pattern recognition. 2017. pp. 2117–25.

[26] Chaurasia A, Culurciello E. Linknet: Exploiting encoder representations for efficient semantic segmentation. In: 2017 IEEE Visual Communications and Image Processing (VCIP). IEEE; 2017. pp. 1–4.

[27] He P, Jiao L, Shang R, Wang S, Liu X, Quan D, et al. MANet: Multi-scale aware-relation network for semantic segmentation in aerial scenes. IEEE Trans Geosci Remote Sensing. 2022;60:1–15. doi: 10.1109/tgrs.2022.3179379

[28] Zhao H, Shi J, Qi X, et al. Pyramid scene parsing network. Proceedings of the IEEE conference on computer vision and pattern recognition. 2017. pp. 2881–90.

[29] Liu S, Qi L, Qin H, et al. Path aggregation network for instance segmentation. Proceedings of the IEEE conference on computer vision and pattern recognition. 2018. pp. 8759–68.

[30] https://drive.google.com/file/d/1reHyY5eTZ5uePXMVMzFOq5j3eFOSp50F/view?pli=1

[31] Gong H, Chen J, Chen G, Li H, Li G, Chen F. Thyroid region prior guided attention for ultrasound segmentation of thyroid nodules. Comput Biol Med. 2023;155:106389. doi: 10.1016/j.compbiomed.2022.106389 36812810

[32] Gong H, Chen G, Wang R, et al. Multi-task learning for thyroid nodule segmentation with thyroid region prior. 2021 IEEE 18th international symposium on biomedical imaging (ISBI). IEEE; 2021. pp. 257–61.

[33] https://drive.google.com/file/d/1wwlsEhwfSyvQsJBRjeDLhUjqZh8eaH2R/view

[34] Pedraza L, Vargas C, Narváez F, et al. An open access thyroid ultrasound image database. 10th International symposium on medical information processing and analysis. SPIE; 2015. 9287: 188–193.

